# Novel Mutations in* MLH1* and* MSH2* Genes in Mexican Patients with Lynch Syndrome

**DOI:** 10.1155/2016/5278024

**Published:** 2016-05-10

**Authors:** Jose Miguel Moreno-Ortiz, María de la Luz Ayala-Madrigal, Jorge Román Corona-Rivera, Manuel Centeno-Flores, Víctor Maciel-Gutiérrez, Ramón Antonio Franco-Topete, Juan Armendáriz-Borunda, Erin Hotchkiss, Lucia Pérez-Carbonell, Jennifer Rhees, Clement Richard Boland, Melva Gutiérrez-Angulo

**Affiliations:** ^1^Instituto de Genética Humana, CUCS, Universidad de Guadalajara, Sierra Mojada 950, Colonia Independencia, 44340 Guadalajara, JAL, Mexico; ^2^Trayectoria de Genómica Alimentaria, Universidad de la Ciénega del Estado de Michoacán de Ocampo, Avenida Universidad 3000, Colonia Lomas de la Universidad, 59103 Sahuayo, MICH, Mexico; ^3^Hospital Civil de Guadalajara “Dr. Juan I. Menchaca”, Salvador de Quevedo y Zubieta 750, 44340 Guadalajara, JAL, Mexico; ^4^Departamento de Biología Molecular y Genómica, CUCS, Universidad de Guadalajara, Sierra Mojada 950, Colonia Independencia, 44340 Guadalajara, JAL, Mexico; ^5^GI Cancer Research Laboratory, Baylor University Medical Center, 3500 Gaston Avenue, Suite 250 Hoblitzelle, Dallas, TX 75204, USA; ^6^Departamento de Clínicas, CUAltos, Universidad de Guadalajara, Carretera a Yahualica Km. 7.5, 47600 Tepatitlán de Morelos, JAL, Mexico

## Abstract

*Background*. Lynch Syndrome (LS) is characterized by germline mutations in the DNA mismatch repair (*MMR*) genes* MLH1*,* MSH2*,* MSH6,* and* PMS2*. This syndrome is inherited in an autosomal dominant pattern and is characterized by early onset colorectal cancer (CRC) and extracolonic tumors. The aim of this study was to identify mutations in* MMR* genes in three Mexican patients with LS.* Methods*. Immunohistochemical analysis was performed as a prescreening method to identify absent protein expression. PCR, Denaturing High Performance Liquid Chromatography (dHPLC), and Sanger sequencing complemented the analysis.* Results*. Two samples showed the absence of nuclear staining for MLH1 and one sample showed loss of nuclear staining for MSH2. The mutations found in* MLH1* gene were c.2103+1G>C in intron 18 and compound heterozygous mutants c.1852_1854delAAG (p.K618del) and c.1852_1853delinsGC (p.K618A) in exon 16. In the* MSH2* gene, we identified mutation c.638dupT (p.L213fs) in exon 3.* Conclusions*. This is the first report of mutations in MMR genes in Mexican patients with LS and these appear to be novel.

## 1. Introduction

Lynch Syndrome (LS), previously called hereditary nonpolyposis colorectal cancer (HNPCC), was described in the early twentieth century by A. S. Warthin and was further characterized in the second half of the twentieth century by Lynch [1]. This syndrome is inherited as an autosomal dominant pattern and is characterized by early onset CRC and other specific extracolonic tumors [2]. Most patients with LS have heterozygous mutations in* MLH1*,* MSH2*,* MSH6,* or* PMS2*, the principal genes in the DNA mismatch repair (MMR) system [3, 4]. The proteins encoded by* MMR* genes are involved in the identification and repair of errors occurring during S phase, and repetitive sequences called microsatellites are particularly vulnerable to mutation in the absence of DNA MMR activity [4–6]. The diagnosis of LS is suspected clinically using the Revised Bethesda Guidelines, and a definitive diagnosis requires the identification of mutations in one of the LS associated genes [7]. A large number of unique mutations are found in LS families, and founder mutations are commonly found in relatively isolated populations [8]. The goal of this study was to identify mutations in* MMR* genes in Mexican patients with LS.

## 2. Material and Methods

### 2.1. Patients

**Figure 1:**
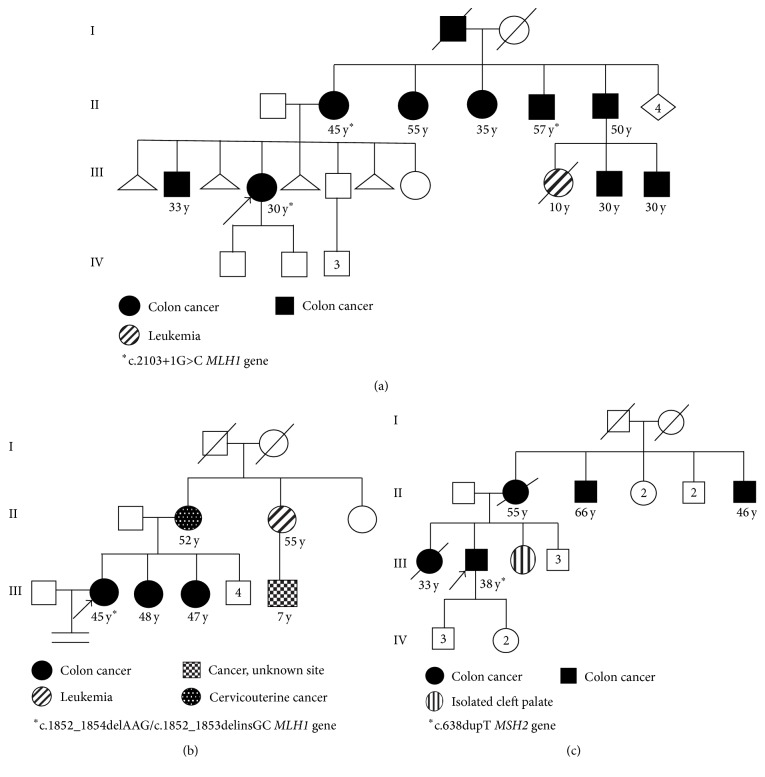
Genealogies from three LS families. (a) Family LS-23, (b) Family LS-41, and (c) Family LS-52.

The study utilized peripheral blood and tumor samples obtained from three unrelated patients diagnosed clinically with LS at the “Dr. Juan I. Menchaca” Civil Hospital of Guadalajara, Jalisco, Mexico (LS-23, LS-41, and LS-52). The genealogies including age and cancers type associated are shown in Figure 1. Two additional family members affected with CRC were analyzed in association with the proband LS-23 (II-2 and II-5). All the patients provided informed consent. The study was performed in accordance with the Helsinki Declaration and considered the ethical aspects of research involving human subjects according to the Mexican General Health Law. This research was approved by the ethics committee of Centro Universitario de los Altos, Universidad de Guadalajara (CUA/CINV/494/2009).

### 2.2. Immunohistochemistry

Protein expression of MLH1, MSH2, MSH6, and PMS2 was analyzed by immunohistochemistry (IHC) staining with the DAKO EnVision System-HRP polymer system kit (DakoCytomation Inc., Carpinteria, CA). The following antibodies were used: for MLH1, clone 13271A, BD Pharmingen, San Diego, CA; for MSH2, clone FE11, Oncogene Research Products, Boston, MA; for MSH6, clone 44, BD Transduction Laboratories, Lexington, KY; and, for PMS2, clone A37, BD Pharmingen. One block of formalin-fixed paraffin-embedded tumor tissue was selected per case. IHC was performed as previously reported [9]. Briefly, 5 *µ*m thick tissues were cut, dewaxed in xylene, and rehydrated in graded alcohol concentrations to buffer. The slides were incubated for one hour with the appropriate dilutions of mouse monoclonal antibodies. The peroxidase reaction was developed using diaminobenzidine tetrachloride as the chromogen. Normal expression of protein was indicated by the presence of nuclear staining in colon cells, and loss of staining in tumor cells was determined only if nonneoplastic colonocytes and stromal cells were positively stained.

### 2.3. DNA Extraction

Genomic DNA was extracted from peripheral blood samples of patients as previously reported [10]. The DNA concentration was measured by spectrophotometry and the quality was evaluated in 1% agarose gel electrophoresis.

### 2.4. dHPLC and DNA Sequencing

Specific primers were used to amplify all 19 exons of* MLH1* gene and 16 exons of* MSH2* gene by polymerase chain reaction (PCR) according to conditions established by the laboratory. The fragments were then visualized by gel electrophoresis in a 2% agarose gel, stained with ethidium bromide.

Denaturing High Performance Liquid Chromatography (dHPLC) was undertaken using the Hitachi WAVE® DNA fragment analysis system HSX-3500 (Transgenomic*™*). An aliquot (5 *µ*L) of the PCR product was directly injected into a DNA Sep column. The samples were analyzed under the optimum melting temperature determined in the GI Cancer Research Laboratory at Baylor University Medical Center, Dallas. The chromatograms of each fragment were compared with those of the wild type, and fragments containing heteroduplexes and shorter retention times compared to wild type fragments underwent Sanger sequencing to confirm putative sequence variations.

PCR products were purified using the QuickStep*™* 2 PCR Purification Kit (EdgeBio, Gaithersburg, MD) and PERFORMA® DTR Gel Filtration Cartridges Kit (EdgeBio). The successive purified products were subjected to cycle sequencing in the forward and reverse directions using a BigDye Terminator v1.1 Kit (Applied Biosystems, Life Technologies) on the automated PRISM 3100-Avant DNA Sequencer (Applied Biosystems, Life Technologies). The sequences for each fragment were analyzed in the genome browser: http://genome.ucsc.edu/.

### 2.5. PCR-RFLP

PCR-RFLP was done for a variant localized in 18 exon-intron boundary of* MLH1 *using standard PCR conditions in samples of family members of proband LS-23 (II-2 and II-5, Figure 1). The primers were as follows: forward 5′-TTT TGA GGT ATT GAA TTT CTT TGG-3′ and reverse 5′-TGA GGT CCT GTC CTA GTC CTG-3′. A PCR fragment of 191 bp was used for digestion with the* Alu*I restriction enzyme. This cuts the mutant allele and the fragments generated for heterozygous individuals were 191 bp, 144 bp, and 47 bp.

## 3. Results

MLH1 and MSH2 protein expression were altered in the tissue samples evaluated. LS-23 and LS-41 showed the absence of nuclear staining for MLH1, and sample LS-52 showed loss of nuclear staining for MSH2.

**Figure 2:**
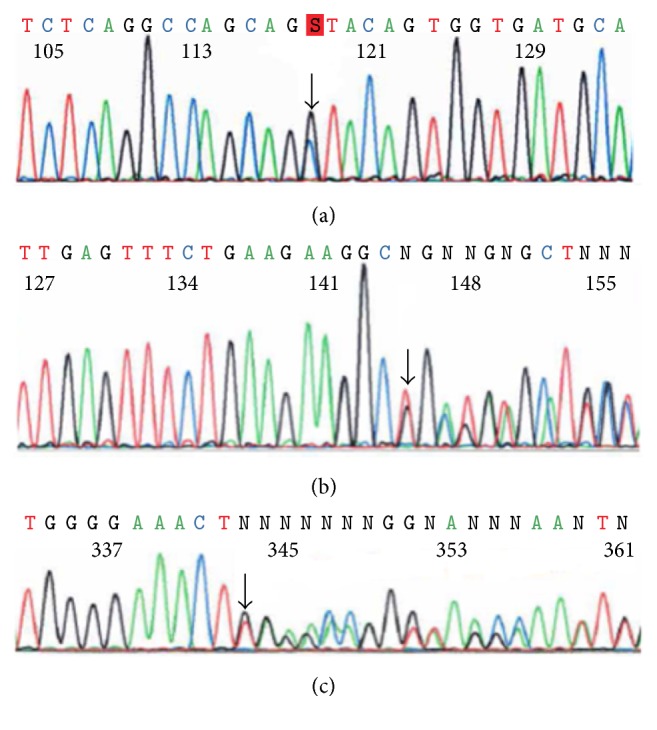
DNA sequencing results in three LS patients. (a) Patient LS-23 with c.2103+1G>C in the* MLH1* gene. (b) Patient LS-41 with two different mutations in the* MLH1* gene: c.1852_1854delAAG (p.K618del) and c.1852_1853delinsGC (p.K618A). (c) Patient LS-52 with mutation c.638dupT in the* MSH2 *gene. The arrows indicate the location of the mutations.

All the exons of* MLH1* and* MSH2* genes were successfully amplified and screened by dHPLC. Samples showing heteroduplex peaks were subjected to DNA sequencing to characterize the germline mutations. Patient LS-23 had a substitution at a splice donor site, located in the first nucleotide base of intron 18 of* MLH1*, c.2103+1G>C. Once we identified the mutation in this patient, two additional members with CRC in this family (II-2 and II-5, Figure 1) were screened by PCR-RFLP as the mutation created a novel restriction site (data not shown). The results showed a heterozygous genotype for the mutation in the analyzed individuals. In patient LS-41, two different sequence variations were found in exon 16 of* MLH1*. One allele had an in-frame deletion of a codon, c.1852_1854delAAG (p.K618del), and the other displayed a deletion/insertion of two bases, c.1852_1853delinsGC (p.K618A), which resulted in a lysine to alanine mutation at codon 618. The third sequence variation was duplication c.638dupT in codon 213 in exon 3 of the* MSH2* gene, in patient LS-52. This resulted in the frameshift mutation, p.L213fs (Figure 2). These data were presented at American Society of Human Genetics Annual Meeting in 2014 [11].

## 4. Discussion

The Revised Bethesda Guidelines recommend MSI analysis and/or IHC analysis of tumor tissue to select patients for definitive DNA sequence analysis [12]. Our study identified three putative LS patients from Mexico, initially suspected based upon family history and screened by IHC and confirmed with DNA analyses. In the initial screening by IHC, we identified two patients with absent expression of* MLH1* and one with absent expression of* MSH2*.

Patient LS-23 had a substitution at a splice donor site, located in the first nucleotide base of intron 18 of* MLH1*, c.2103+1G>C. This change is located in a canonical GT/AG splice site. The phyloP and phastCons values were 4.281 and 1, respectively, suggesting strong evolutionary conservation [13]. This mutation is included in the SNP database as rs267607888 whereas the changes G>A and G>T (c.2103+1G>A and c.2103+1G>T) have been reported in patients with LS in the PubMed database [14–20]. Variations in this position are classified as likely pathogenic or pathogenic by the InSiGHT Variant Interpretation Committee, respectively [21]. Mutation G>C found in this patient (c.2103+1G>C) of* MLH1* has apparently been reported only in the InSiGHT database [21]. The mutational effect on the MLH1 protein is unclear since mutations in splice site sequences could promote exon skipping, intron retention, or activation of cryptic splice sites [22].

Patient LS-41 had a c.1852_1853delinsGC (p.K618A) (rs35502531; c.1852_1853delAAinsGC) variant in one allele and a c.1852_1854delAAG (p.K618del) (rs63751247) sequence variation in the other allele of* MLH1*. The K618A variant is classified as nonpathogenic in the InSiGHT database and is not located in the interaction domain with the PMS2 protein described between amino acids 531–549 and 740–756 in* MLH1* [23]. Thompson et al. used multifactorial likelihood analysis to conclude that c.1852_1853delinsGC is not pathogenic [24]. Moreover, in a recent study published by Abulí et al., this variant was found in 1.4% of 8,055 CRC cases and 1.5% of 10,668 controls from seven cohorts. They concluded that c.1852_1853delinsGC in* MLH1* plays no role in the genetic predisposition to LS [25]. In a multifactorial likelihood analysis by Thompson et al. in 2013 [24], a pathogenic effect was found for p.K618del, which was consistent with a previous report by Takahashi et al. in 2007 [26]. Takahashi et al. performed functional analyses of different variants of the* MLH1* gene, including p.K618A and p.K618del. They reported that p.K618A has an* in vitro* MMR activity of 82.7%, while that for p.K618del was 38.9%. Additionally, reduced* MLH1* expression (<25%) was observed in association with the p.K618del variant [26]. Since most of reports indicate that c.1852_1854delAAG (p.K618del) has a pathogenic effect, this should be considered the sequence variant responsible for LS in Family LS-41. In a segregation analysis reported by Castillejo et al. they concluded that the p.K618A polymorphism should be considered a neutral variant [27], although they do not exclude the variant as a possible susceptibility factor in CRC development. Moreover, the expression assays demonstrated that p.K618A compromises protein stability and decreases the half-life of MLH1 protein by a factor of 2.5–3.5 [28]. Perhaps the compound heterozygous variants (c.1852_1853delinsGC and c.1852_1854delAAG) in* MLH1* in patient LS-41 synergize to predispose to the CRC.

Patient LS-52 had a duplication of a single nucleotide, c.638dupT, which resulted in the frameshift mutation p.L213fs in the* MSH2* gene. An* in silico* analysis of this duplication, using the CCDS1834 sequence (*MSH2* gene) and the molecular toolkit program from Colorado State University (http://www.vivo.colostate.edu/) [29], indicated that the duplication of T generates a protein of 230 amino acids instead of the wild type protein of 934 amino acids, due to the premature stop in codon 231. Mangold et al. analyzed 1721 German patients and detected a frameshift mutation in three patients in this specific region; however the alteration in the DNA coding sequence was different, c.638_639delTG [30]. Although frameshift mutations in codon 213 of exon four of* MSH2* gene have been described previously [30, 31], the c.638dupT alteration has not been reported in any LS database, including the International Society for Gastrointestinal Hereditary Tumors Mutation Database (http://insight-group.org/) [21], the Human Gene Mutation Database (http://www.hgmd.cf.ac.uk/ac/index.php) [32], or the MMR Genes Variant Database (http://www.med.mun.ca/variants) [33]. Therefore, this is likely a novel mutation.

LS is the most common form of inherited CRC and accounts for about 3% of all CRC cases [34]. In Mexico, CRC has a lifetime incidence of 5.8% according to Globocan 2012 [35]. The search for* MMR* genes mutations in CRC patients is essential for making the diagnosis in LS families [1]. The individuals positive for these mutations will have a surveillance according to international guidelines [36] in order to avoid the cancer development or progression.

## 5. Conclusions

This study reports the c.638dupT in* MSH2* and the compound heterozygous alteration c.1852_1853delinsGC/c.1852_1854delAAG in* MLH1* as novel findings in LS. Moreover, these findings highlight the importance of identifying novel genetic alterations populations that have not been previously studied.
